# Advances in Diagnostic Testing for Eosinophilic Esophagitis: Current Approaches, Limitations, and Emerging Technologies

**DOI:** 10.3390/diagnostics16203287

**Published:** 2026-10-10

**Authors:** Maanasa Naga Laxmi Pulavarty, Amy Meehan, Cary Qualia

**Affiliations:** 1 Albany Medical College, Albany, NY 12208, USA; 2 Department of Pediatric Gastroenterology, Albany Medical Center, Albany, NY 12208, USA; meehana2@amc.edu (A.M.); qualiac@amc.edu (C.Q.)

**Keywords:** eosinophilic esophagitis (EoE), dysphagia, non-invasive diagnostic tools

## Abstract

Eosinophilic esophagitis (EoE) is a chronic disease of the esophagus caused by an abnormal type 2 (Th2) immune response. It is a leading cause of dysphagia in both pediatric and adult populations. Endoscopy with biopsy remains the standard approach for diagnosis, but its invasive nature and need for repeated procedures have prompted investigation of alternative and complementary approaches. A narrative review was conducted to synthesize evidence on established and emerging modalities for EoE diagnosis and disease assessment, including minimally invasive sampling techniques, functional and structural assessment, mucosal impedance, molecular diagnostics, tissue-based assessment, and artificial intelligence (AI)-assisted approaches. Minimally invasive approaches may facilitate diagnosis or longitudinal monitoring, while complementary modalities provide assessment of structural, functional, histologic, and molecular features of disease. In retrospective image datasets, AI models have demonstrated high performance for automated eosinophil quantification and detection f endoscopic features, although none has been prospectively validated in routine clinical practice. Despite these advances, widespread adoption remains limited by cost, accessibility, and variability in validation across diverse populations. Further prospective and external validation is needed to establish the role of these modalities in mainstream practice.

## 1. Introduction

Eosinophilic esophagitis (EoE) is a chronic, type 2 (Th2) immune-mediated disease characterized by symptoms of esophageal dysfunction and eosinophil-predominant inflammation on histology [1,2]. Since its recognition as a distinct clinical condition, EoE has emerged as a leading cause of dysphagia and food impaction among children and young adults [1].

The significant rise in incidence and prevalence of EoE over the past two decades cannot be attributed to increased disease awareness alone. A meta-analysis of population-based studies estimated the pooled adult incidence at 7.7 per 100,000 person-years and prevalence at 42.2 per 100,000 persons [3]. Pooled prevalence has also risen from 8.18 (1976–2001) to 74.42 (2017–2022) per 100,000 persons [4]. Clinical presentation varies by age, ranging from feeding intolerance and failure to thrive in pediatric populations to dysphagia and esophageal food impaction in adults [1]. Diagnostic delay remains a challenge, with a median delay of 4 years and approximately one third of patients undiagnosed for 10 years or longer [5]. Each additional year of untreated symptoms is also associated with an approximately 9% increase in the odds of stricture development, emphasizing the need for early and accurate diagnosis [2,6].

Current consensus guidelines, most recently reinforced by the 2025 American College of Gastroenterology (ACG) Clinical Guideline, define EoE by three criteria: (1) symptoms of esophageal dysfunction, (2) ≥15 eosinophils per high-power field (eos/hpf) on esophageal biopsy, and (3) evaluation for non-EoE disorders that cause or potentially contribute to esophageal eosinophilia [1]. Although these criteria have standardized diagnosis, challenges remain due to the nonspecific nature of symptoms and variability in clinical presentation.

While endoscopy with biopsy remains the standard for diagnosis, this approach is invasive, resource-intensive, and subject to sampling variability due to the patchy distribution of eosinophilic infiltration [1]. Histologic findings may not correlate well with symptom severity, and repeated testing imposes a significant burden on patients and healthcare systems [7]. These limitations have driven the development of functional assessment tools, minimally invasive sampling devices, biomarkers, and artificial intelligence-driven analysis [1,7]. This paper provides an overview of current and emerging diagnostic approaches in EoE, highlighting their utility, limitations, and potential impact on future diagnostic algorithms.

## 2. Methodology

A narrative literature review was conducted to evaluate established and emerging approaches to the diagnosis and assessment of eosinophilic esophagitis (EoE), with particular emphasis on evolving diagnostic technologies and artificial intelligence–based approaches. The literature reviewed spanned publications from 2009 through 2026 and was identified using PubMed, with additional articles identified through the references of key publications, guidelines, and consensus statements. Search terms included combinations of “eosinophilic esophagitis,” “EoE,” “diagnosis,” “diagnostic,” “biomarker,” “endoscopy,” “histology,” “artificial intelligence,” and “disease monitoring.” Articles were selected based on their relevance to the diagnostic evaluation of EoE, including established diagnostic modalities, emerging noninvasive and minimally invasive approaches, molecular and histologic assessment, and artificial intelligence–assisted diagnosis. For the purpose of this review, noninvasive approaches were defined as those that do not require esophageal instrumentation or tissue acquisition. Minimally invasive approaches involved limited esophageal instrumentation or sampling without conventional sedated esophagogastroduodenoscopy (EGD). Emphasis was placed on studies reporting diagnostic performance, disease activity assessment, treatment response, validation, or clinical applicability. Primary studies were prioritized when available, particularly for reporting diagnostic accuracy and validation data, while guidelines, consensus statements, and relevant reviews were included to provide clinical and historical context. The second author reviewed the selected references and reported study findings for accuracy and consistency with the cited literature. As a narrative review, no formal systematic-review protocol or meta-analysis was performed.

## 3. Conventional Diagnostic Evaluation of EoE

### 3.1. Major Consensus and Guidelines

The diagnostic model for EoE has evolved since the first guidelines were established in 2007. The most current guidance comes from the 2025 American College of Gastroenterology (ACG) Clinical Guideline, the 2022 American Society for Gastrointestinal Endoscopy (ASGE) consensus conference, and the 2018 AGREE (A Working Group on PPI-REE) international consensus conference [1,7,8]. Guidelines from United European Gastroenterology and the European Society of Eosinophilic Esophagitis have also contributed to standardizing diagnostic criteria internationally [9].

A major change in the diagnostic approach was the elimination of the requirement for a proton pump inhibitor (PPI) trial prior to diagnosis and the subsequent removal of the term PPI-responsive esophageal eosinophilia (PPI-REE). Data demonstrated that patients who responded to PPIs were clinically, endoscopically, and histologically similar to patients without PPI response. Furthermore, PPIs likely treat EoE through anti-inflammatory mechanisms independent of gastric acid suppression alone [1]. This change was formalized in the 2018 AGREE conference and reinforced by the 2025 ACG guideline.

### 3.2. Diagnostic Criteria

EoE is diagnosed based on three criteria: (1) symptoms of esophageal dysfunction; (2) at least 15 eosinophils per high-power field (eos/hpf) on esophageal biopsy; and (3) evaluation for non-EoE disorders that lead to esophageal eosinophilia [1,2]. While the exact eosinophil count does not directly reflect disease severity, the 2025 ACG guideline recommends quantifying eosinophil counts on each endoscopic biopsy to gauge the anti-inflammatory effect of treatment. To account for variability due to high-power field size, eosinophil density may also be reported to allow comparisons across different clinical settings.

### 3.3. Clinical Presentation

Clinical presentation varies with age. Adults with EoE commonly present with dysphagia, food impaction, heartburn, and chest pain [1,2]. It is estimated that almost 50% of adult patients diagnosed with EoE initially present to the Emergency Department with a food impaction [10]. Additional symptoms that may raise suspicion for EoE include repetitive vomiting after food consumption, regurgitation, and epigastric pain. Younger children often present with food refusal and poor growth, prompting further evaluation for EoE [10]. EoE is also strongly associated with atopic conditions such as asthma, eczema, and allergies [1]. A history of IgE-mediated food allergies or a family history of EoE may further increase suspicion in patients with compatible symptoms.

### 3.4. Endoscopy and Histologic Confirmation

EGD with esophageal biopsies remains the standard diagnostic approach for EoE. In addition to histologic findings, the Eosinophilic Esophagitis Endoscopic Reference Score (EREFS) is widely used to standardize the assessment of endoscopic disease activity and monitor treatment response [7]. EREFS assesses the five most commonly observed endoscopic findings in patients with EoE (edema, rings, exudates, furrows and strictures). In the original system, edema and furrows were graded across multiple severity levels. However, poor interobserver agreement for edema led to a modified system in which the severity grading for both edema and furrows was collapsed. In this modified system, edema and furrows are scored as absent or present (0–1), while rings, exudates, and strictures are scored on scales of 0–3, 0–2, and 0–1, respectively [11]. The modified system demonstrated good interobserver agreement for the major endoscopic features of EoE and is consistent with the simplified EREFS scoring recommended for clinical use in ASGE guidance [7].

The modified EREFS scoring system has demonstrated good discriminatory ability between patients with EoE and controls. In the validation study, the mean total EREFS was 3.88 among patients with EoE compared with 0.42 among controls, with an AUROC of 0.934 [12]. Histologic confirmation requires adequate esophageal sampling because eosinophilic infiltration can vary in distribution throughout the esophagus. At least six esophageal biopsies should be obtained, including samples from the distal esophagus and from either the mid or proximal esophagus [7]. Areas of visible inflammation should be targeted when possible. However, biopsies should be obtained whenever EoE is suspected, regardless of the gross appearance of the esophagus, as the esophagus may appear grossly normal in approximately 10–32% of adult and pediatric patients at the time of diagnosis [7].

Other causes of esophageal eosinophilia should also be considered and evaluated when appropriate. These include gastroesophageal reflux disease, hypereosinophilic syndrome, connective tissue diseases, Crohn disease, infections, pill-induced esophagitis, and autoimmune disease [2].

### 3.5. Limitations of Conventional Diagnosis

Although endoscopic biopsy provides a reliable means of establishing the diagnosis of EoE, conventional endoscopic evaluation has several limitations. The need for EGD and histologic sampling makes diagnosis invasive and may require anesthesia or sedation, which is particularly relevant in young children and older adults. Repeat endoscopies are often required to evaluate histologic response to therapy, increasing procedural burden and repeated exposure to anesthesia or sedation. Esophageal perforation is a rare but serious complication of endoscopy and biopsy, while bleeding represents an additional procedural risk [7]. Histologic sampling may introduce additional limitations because inadequate sampling, including failure to obtain biopsies from multiple esophageal locations, can result in missed disease [7]. Peak eosinophil density remains the principal histologic endpoint for diagnosis and assessment of treatment response, but eosinophil counts do not consistently correlate with symptom severity [1]. Furthermore, eosinophil density alone does not capture other features of EoE such as epithelial injury, basal zone hyperplasia, or fibrotic remodeling. These limitations have prompted investigation of less invasive approaches for EoE diagnosis and disease monitoring, including blood-based, esophageal secretion-based, imaging-based, and molecular-based modalities.

## 4. Emerging and Advancing Diagnostic Modalities

### 4.1. Alternatives to Conventional Endoscopy

#### 4.1.1. Unsedated Transnasal Endoscopy

Unsedated transnasal endoscopy (TNE) is an office-based, anesthesia-free procedure in which an ultrathin flexible endoscope or small-caliber bronchoscope is passed through a topically anesthetized nasal passage into the esophagus. TNE permits direct visualization of the esophageal mucosa and acquisition of esophageal biopsies without sedation or general anesthesia [13]. The 2025 ACG guideline recognizes unsedated TNE as a safe and well-tolerated approach, particularly in children, that can provide both endoscopic and histologic measures of treatment response [1].

In a prospective pilot study of 21 children, TNE biopsies provided epithelial surface areas comparable to those obtained during prior EGD, with no serious adverse events. Most parents and approximately half of children preferred TNE over EGD, and procedural charges were 60.1% lower [14]. A retrospective study of 190 children and young adults undergoing 294 TNE procedures demonstrated a 98% completion rate, with full-thickness epithelium obtained in 88–94% of TNE biopsies compared with 89% of EGD biopsies. Use of TNE was associated with approximately 53% lower charges than EGD, largely because of avoided anesthesia costs [15]. In a more recent matched comparative study of 66 TNE procedures with 132 EGDs, TNE reduced time spent in the gastrointestinal suite by approximately 1.9 h while demonstrating comparable rates of visual endoscopic findings and most components of the Eosinophilic Esophagitis Histology Scoring System (EoEHSS) [16].

Evidence in adults is more limited but suggests that TNE can also provide adequate histologic sampling in selected patients. In a prospective feasibility study of 24 adults with EoE who underwent 74 procedures, nasal intubation was successful in 97% of procedures and all completed procedures yielded adequate histologic samples. 86% of participants reported high satisfaction and would choose TNE again [17]. However, only 24 of 96 adults offered TNE elected to participate, highlighting potential selection and acceptability considerations when expanding these findings to broader adult populations.

A consistent limitation of TNE is that the small caliber of the biopsy forceps reduces lamina propria yield and consequently limits fibrosis grading. While this is partly mitigated by taking additional biopsies, this remains a technical trade-off compared to EGD [15]. TNE also does not permit therapeutic interventions such as esophageal dilation. These considerations, paired with the limited evidence base in adults, currently position TNE as a complementary approach for serial disease surveillance rather than a replacement for conventional EGD. The ability to obtain both endoscopic and histologic measures without sedation makes TNE an attractive strategy for reducing the cumulative procedural burden of long-term EoE monitoring [14].

#### 4.1.2. Cytosponge

The Cytosponge is a minimally invasive sampling device consisting of a mesh sponge compressed in a gelatin capsule and attached to a string. Within minutes of ingestion, the capsule dissolves, allowing the sponge to expand in the esophagus. The device is then withdrawn and collects mucosal tissue along its surface for subsequent fixation and histologic analysis [2].

While standard endoscopic biopsy targets discrete areas of the esophagus, the Cytosponge samples a broader segment of mucosa. This wider coverage may improve detection of patchy eosinophilic inflammation. In a prospective two-center study of 80 EoE patients, 101 procedures had adequate paired Cytosponge and endoscopic biopsy sampling. Eosinophil counts demonstrated strong correlation between the two methods (r = 0.78, *p* < 0.0001). Using ≥15 eos/hpf on endoscopic biopsy to define active EoE, Cytosponge demonstrated 75% sensitivity and 86% specificity for detecting histologic disease activity [18]. These findings support the feasibility of Cytosponge for histologic activity assessment in patients with established EoE.

The Cytosponge procedure is generally well tolerated and does not require sedation. These features make it attractive for longitudinal monitoring and for use in food reintroduction protocols that necessitate repeated assessments. Despite these advantages, several limitations should be considered. A sensitivity of 75% indicates that a subset of patients with active disease may be missed. Furthermore, the study cohort was 100% White, which may limit the generalizability of these findings to more diverse populations [18]. Successful administration of the device depends on the patient’s ability to swallow the capsule, which may be limited in patients with significant dysphagia. Notably, 7% of patients in the prospective study were unable to swallow the device [18]. The test also requires histologic processing similar to that of standard biopsy.

Overall, the Cytosponge represents a minimally invasive approach to mucosal assessment in EoE. Although current evidence does not support complete replacement of endoscopic evaluation, it may serve as a practical adjunct for disease monitoring and could reduce the need for repeated sedated procedures.

#### 4.1.3. Esophageal String Test

The esophageal string test (EST) is a minimally invasive device designed to sample luminal contents of the esophagus. Once swallowed, the absorptive string unravels and remains in place for approximately one hour to absorb esophageal secretions. The string is then withdrawn for analysis of inflammatory biomarkers, allowing for the assessment of disease activity without histologic evaluation [1].

Several studies have demonstrated its ability to distinguish active from inactive disease. In patients with EoE, measurement of eotaxin-3 and major basic protein-1 demonstrated a strong discriminatory ability between active (≥15 eos/hpf) and inactive (<15 eos/hpf) disease, with an AUC of 0.83 [19]. In a cohort of 134 patients, a statistical model combining eotaxin-3 and major basic protein-1 generated EoE scores that distinguished active EoE from inactive disease or normal esophagus, achieving 80% sensitivity and 75% specificity [19].

The EST is also a promising tool for treatment monitoring as it allows for repeated assessment of disease activity without the need for sedated endoscopy. A systematic review further supported its role, demonstrating strong correlations between non-biopsy esophageal sampling methods and histologic eosinophil counts. These associations were most apparent when evaluating eosinophil-derived proteins [20].

A major limitation of EST is that it does not provide tissue for histologic analysis, which remains central to the diagnosis of EoE. Furthermore, results depend on patient compliance during the procedure and require specialized laboratory processing of biomarkers. Overall, the EST offers a less invasive approach to assessing esophageal inflammation in EoE and may serve as a useful adjunct for monitoring disease activity and guiding management over time.

#### 4.1.4. Unsedated Tethered Capsule Endomicroscopy

Unsedated tethered capsule endomicroscopy (TCE) is an emerging imaging modality that uses a swallowable capsule coupled to a flexible tether to obtain cellular-resolution images of the esophageal mucosa without sedation or biopsy. TCE uses spectrally encoded confocal microscopy to visualize microscopic features such as intraepithelial eosinophils and their bilobed nuclei. In a first-in-human pilot study of 17 subjects, including 13 patients with EoE and 4 healthy controls, the capsule was successfully swallowed in 16 of the 17 subjects. Among these patients, TCE successfully acquired cellular-resolution images of the mucosa, with representative cases demonstrating agreement between TCE findings and biopsy histology. The device provided broad coverage of the esophageal mucosa, with a mean imaged tissue area equivalent to approximately 7000 conventional microscopic high-power fields [21]. Current evidence is limited to a small feasibility study without formal diagnostic performance measures, and further studies are needed to establish diagnostic accuracy and clinical utility.

#### 4.1.5. Blood and Airway Biomarkers

Considerable effort has been directed toward identifying blood-based biomarkers for EoE, but no single marker has demonstrated consistent diagnostic accuracy. A systematic review of over 80 proposed non-invasive biomarkers found that peripheral eosinophil count has limited reliability for diagnosis and disease monitoring despite extensive research [20].

More recent prospective data suggest slight improvement with refined measures. In a multicenter study of 209 patients, absolute eosinophil count (AEC) distinguished EoE from non-EoE dysphagia with an AUC of 0.85 (95% CI, 0.788–0.908). At a cutoff of 155 eosinophils/mm^3^, AEC demonstrated 87% sensitivity and 71% specificity. For assessment of histologic disease activity, AEC discriminated active EoE from histologic remission with an AUC of 0.705 (95% CI, 0.612–0.798). At a cutoff of 325 eosinophils/mm^3^, AEC demonstrated 52.7% sensitivity and 82.3% specificity for distinguishing active disease from histologic remission [22]. Despite its diagnostic performance, the authors reported that most patients with EoE had AEC values within the normal laboratory range, which may limit its utility as a stand-alone diagnostic test [22].

Greater accuracy has been achieved using multi-marker approaches. Panels incorporating various eosinophil-derived proteins have consistently outperformed AEC alone in distinguishing EoE from non-EoE controls [23]. In a pediatric cohort, a combined model of AEC, eosinophil-derived neurotoxin (EDN), food-specific IgE, and clinical symptoms achieved an AUC of 0.92 in differentiating active disease, remission, and healthy states [24]. Several of these markers also decrease with effective disease treatment. Serum EDN, AEC, and eosinophil cationic protein have been shown to decrease in patients achieving histologic remission, supporting their potential role in longitudinal disease monitoring [23].

Other emerging approaches include fractionated exhaled nitric oxide (FeNO) and metabolomic profiling. In a prospective, single-center cohort of 62 patients, FeNO levels were higher in patients with active EoE compared with those with GERD (median 20 ppb vs. 15 ppb, *p* = 0.038) and healthy controls (14 ppb, *p* = 0.046). A FeNO cutoff of >15 ppb demonstrated 75.9% sensitivity and 51.6% specificity for distinguishing active EoE from non-EoE patients. However, FeNO did not significantly change following treatment response (20 vs. 18 ppb, *p* = 0.771), limiting its utility for longitudinal disease monitoring [25].

More recently, a prospective cohort study of 128 children undergoing endoscopy for suspected esophageal or peptic disease found that a multimarker score incorporating FeNO, β-alanine, cysteine, and spermine demonstrated an AUC of 0.90 (95% CI, 0.80–0.96) for distinguishing active EoE from controls [26].

Despite these advances, important limitations remain. No individual biomarker has demonstrated sufficient sensitivity or specificity for clinical use, and performance varies considerably across studies. Many panels have been validated only in pediatric populations, and results may be confounded by coexisting atopic disease. Blood-based biomarkers currently remain investigational but represent a promising direction for future development in both diagnosis and longitudinal monitoring.

### 4.2. Complementary Diagnostic Assessment

#### 4.2.1. Manometry

High-resolution manometry (HRM) has been explored as a potential tool for evaluating esophageal dysfunction in EoE, but its diagnostic role remains limited. A scoping review of 802 patients found that the overall prevalence of motility abnormalities in EoE was similar to that seen in other populations undergoing manometry for esophageal symptoms. One notable exception was esophagogastric junction outflow disorders, which were found in approximately 5% of patients [27].

One of the more characteristic findings of EoE is panesophageal pressurization, a pattern defined by simultaneous pressurization of the esophageal body rather than coordinated peristalsis. This pattern is the most commonly reported HRM abnormality and can help raise clinical suspicion for EoE [28]. In a comparative study of 21 adults with EoE and 21 controls with GERD symptoms and dysphagia, panesophageal pressurization was observed in 48% of patients and was associated with food bolus impaction [29]. Prolonged esophageal manometry has also demonstrated that symptoms of dysphagia in EoE correlate sequentially with abnormal motor events, including ineffective peristalsis and high-amplitude contractions [30].

A subset of these motility issues was also found to improve with anti-inflammatory therapy, suggesting that esophageal dysfunction in EoE is at least partially driven by inflammation rather than fixed structural changes [27]. Despite this, HRM findings remain nonspecific and correlate poorly with both symptom severity and endoscopic features [31].

HRM has not been incorporated into standard diagnostic criteria for EoE and is reserved for select clinical scenarios such as persistent dysphagia or suspicion of a coexisting primary motility disorder [28]. Its limited diagnostic utility reflects continued reliance on histologic and endoscopic assessment for EoE diagnosis, with functional testing reserved for select clinical scenarios.

#### 4.2.2. EndoFLIP (Functional Lumen Imaging Probe)

The EndoFLIP measures esophageal distensibility and biomechanical variables during sedated endoscopy through impedance planimetry. In EoE, chronic inflammation can progress to fibrosis, resulting in luminal narrowing and loss of mural compliance [1]. These biomechanical changes are thought to contribute substantially to clinical symptoms but are often underestimated on standard endoscopy [32]. A key EndoFLIP metric is the distensibility plateau, defined as the static esophageal diameter despite increasing distention volume and pressure. Reduced distensibility (distensibility plateau < 225 mm^2^, diameter ~17 mm) has been associated with an increased risk of future food impaction [33].

FLIP panometry allows for biomechanical phenotyping of EoE. A PhysioMechanical classification system has identified seven distinct EoE phenotypes based on distensibility, compliance, esophagogastric junction diameter, and contractile response patterns [34]. In addition to characterizing disease phenotype, FLIP-derived metrics, such as C2D2 (a composite measure of esophageal compliance, contractile response, distensibility plateau, and diameter), may help identify patients who are more likely to respond to specific therapies. In a cohort of 46 patients off treatment at baseline, a C2D2 score ≤ 3 was associated with future histologic response to PPI therapy (OR 14.5, 95% CI 2.6–85), with a wide confidence interval reflecting the limited sample size [35]. The 2025 AGA Clinical Practice Update recommends considering FLIP when the mechanism of persistent dysphagia in patients with treated EoE is not apparent on high-quality upper endoscopy or histopathology [36].

In a retrospective, single-center series of 109 patients with dysphagia, 25 of the 29 patients with EoE (86.2%) undergoing EndoFLIP demonstrated reduced distensibility. Among these patients, reduced distensibility was associated with treatment escalation, including dilation or biologic therapy [37]. EndoFLIP can detect reduced esophageal distensibility even in patients without endoscopically visible strictures, suggesting that subclinical fibrotic remodeling may be present despite the absence of obvious structural abnormalities [1]. Current data collectively suggest that assessing esophageal biomechanics provides information not captured by endoscopy and histology alone.

#### 4.2.3. Mucosal Impedance

Mucosal impedance (MI) is an endoscopic technique used to assess esophageal epithelial integrity. Patients with EoE exhibit a characteristic MI pattern with uniformly low impedance values along the entire esophagus. The lower values reflect impaired epithelial integrity and increased mucosal permeability. This pattern differs from GERD, where low values are found only in the distal esophagus [38].

Diagnostic performance has been strong in early studies. A pattern-based MI approach identified EoE with 100% sensitivity and 96% specificity in a validation cohort of 49 patients [38]. Given the relatively small cohort size, the precision of these estimates is limited. Similarly, a balloon MI catheter system demonstrated an AUC of 0.89 for identifying EoE in a development cohort of 69 patients that included GERD and non-GERD groups [39]. Threshold-based analysis has also been explored in a study of 30 patients, including 10 patients in each cohort of active EoE, inactive EoE, and controls. An MI value below 2300 Ω identified active disease with 90% sensitivity and 91% specificity. These values correlate inversely with eosinophil counts and dilated intracellular spaces [40].

MI provides a rapid and objective assessment of mucosal integrity. Measurements can be obtained in minutes and can help distinguish EoE from GERD. However, variability in readings within individual patients and limited availability currently restricts its broader application [41]. Overall, mucosal impedance represents a promising adjunct in the evaluation of EoE. While it offers real-time functional information, its role remains complementary to established diagnostic methods.

### 4.3. Tissue-Based Assessment

Tissue-based assessment in EoE is expanding beyond eosinophil quantification to include a broader range of inflammatory and structural cell types. The 2025 ACG guideline highlights growing evidence implicating mast cells, multiple T-cell subsets, basophils, natural killer cells, and fibroblasts in the disease process [1]. This expanded framework reflects growing recognition that EoE involves a broader inflammatory and remodeling response beyond eosinophilic inflammation alone.

Further support for this shift comes from clinical trials of eosinophil-depleting therapies. Although these treatments effectively reduce tissue eosinophil counts, many patients continue to experience persistent symptoms and endoscopic abnormalities [1]. This suggests that eosinophil depletion alone may be insufficient for complete disease control.

#### 4.3.1. Eosinophilic Esophagitis Histology Scoring System

To address this histologic complexity, the Eosinophilic Esophagitis Histology Scoring System (EoEHSS) was developed. The EoEHSS evaluates eight histologic features for severity (grade) and extent (stage), including eosinophil density, basal zone hyperplasia, eosinophil abscesses, eosinophil surface layering, dilated intercellular spaces, surface epithelial alteration, dyskeratotic epithelial cells, and lamina propria fibrosis. In the initial validation study of 201 biopsy samples from 104 subjects, the EoEHSS demonstrated moderate to strong interobserver agreement among three pathologists and significantly higher grade and stage scores in untreated compared with treated patients. Notably, features independent of eosinophil counts, including basal zone hyperplasia and dilated intercellular spaces, were associated with treatment status. In logistic regression models, EoEHSS-derived principal components demonstrated greater discrimination of treatment status than distal peak eosinophil count (AUC 77.8 vs. 69.8) [42]. While the scoring system was also reported to be usable by pathologists after minimal training, its complexity and limited clinical adoption compared with conventional eosinophil quantification currently favor its use as a complementary rather than a replacement measure in routine practice.

Overall, tissue-based assessment in EoE is becoming more comprehensive, incorporating measures of inflammation and remodeling beyond eosinophil density. This shift reflects growing recognition that disease activity is not fully captured by eosinophil counts alone.

#### 4.3.2. Molecular Approaches

The EoE Diagnostic Panel (EDP) is a 96-gene quantitative PCR array that differentiates EoE patients from healthy controls with high accuracy (96% sensitivity and 98% specificity) [43]. The assay can be performed on both fresh and formalin-fixed, paraffin-embedded tissue samples. Beyond diagnosis, it has been used to identify patients in histologic remission, those exposed to swallowed corticosteroids, and individuals at increased risk of relapse [43].

A key advantage of the EDP is its ability to address sampling variability inherent to conventional histology. In a multicenter study of 507 esophageal biopsy samples from patients with EoE, a single distal esophageal biopsy analyzed with the EDP predicted proximal esophagitis with 85% sensitivity. In cases where histology and molecular results differ, a positive EDP despite histologic remission was associated with an 11-fold increase in the odds of EoE relapse over two years [44]. These findings suggest that molecular profiling may detect subclinical disease activity not captured by routine histologic assessment.

Molecular endotyping has further refined disease classification beyond eosinophil counts. Three EoE endotypes have been identified through molecular and clinical analysis. EoEe1 represents a mild phenotype with gene expression profiles closer to healthy tissue. EoEe2 is characterized by prominent type 2 inflammatory signaling and a steroid-refractory phenotype. EoEe3 is associated with a narrow-caliber esophagus, severe disease, and reduced expression of epithelial differentiation pathways [45]. These endotypes were independent of peak eosinophil counts and demonstrated stability across various analytical methods [45].

Further molecular heterogeneity has been identified within type 2 inflammatory signaling. Five molecular subgroups have been identified, differing in age distribution and transcriptional profiles but not in eosinophil density. This reinforces the concept that similar histologic disease can reflect distinct underlying immune mechanisms and supports potential applications in molecularly guided precision therapy [46].

Transcriptomic profiling has also shown demographic influences on gene expression. Bulk RNA sequencing studies revealed age- and sex-related differences in the EoE transcriptome. Pediatric patients demonstrated relatively greater mast cell signatures, whereas adults showed increased macrophage-associated pathways. Distinct transcriptional differences were also found between male and female patients, suggesting biologic heterogeneity beyond histologic severity [47].

Overall, molecular approaches provide a framework for precision medicine in EoE. While these tools offer high diagnostic accuracy and improved disease stratification, their clinical use remains largely investigational and limited to specialized research settings.

### 4.4. Artificial Intelligence and Digital Pathology

The applications of AI in EoE diagnosis have expanded across histologic, endoscopic, and molecular fields.

#### 4.4.1. Automated Eosinophil Quantification

Several convolutional neural network (CNN)-based models have been developed for automated eosinophil detection and quantification on whole-slide images (WSIs). These approaches complement expert pathologists in assessing peak eosinophil counts. In a study of 203 patients with mucosal eosinophilia, a CNN-based model showed high correlation with pathologist assessments (Pearson r = 0.89–0.91; ICC = 0.83–0.87), comparable to inter-pathologist agreement (r = 0.88; ICC = 0.91). The same model accurately identified peak eosinophil counts > 15 eosinophils/high-power field with an AUC of 0.98 when compared with pathologist assessments and also demonstrated similar performance to pathologists in scoring several EoEHSS features [48].

Additional platforms have reported similar performance. The Open-EoE toolkit incorporates three deep learning models with ensemble learning. In a dataset of 289 WSIs from 183 pediatric patients, the model’s predicted peak eosinophil counts were compared with pathologist assessments from Vanderbilt University Medical Center. At the diagnostic threshold of ≥15 eosinophils/high-power field, the toolkit achieved 91% accuracy [49].

Another CNN-based approach utilized a multi-label segmentation network to identify and quantify both intact and non-intact eosinophils on WSIs. The model was evaluated on 1066 images from 400 patients enrolled in the CEGIR/OMEGA longitudinal cohort study, a multicenter study designed to characterize the natural history of eosinophilic gastrointestinal diseases during routine clinical care. The model achieved a global accuracy of 94.75%, with 94.13% sensitivity and 95.25% specificity for reporting EoE disease activity [50].

Beyond quantification, newer AI models are extending into spatial and architectural analysis. One platform used semantic segmentation to analyze the distribution of eosinophils and basal zone fractions across WSIs. Using the same CEGIR/OMEGA cohort of 1066 images, the model classified histologic disease severity with 86.70% accuracy, 84.50% sensitivity, and 90.09% specificity [51].

These advances suggest a shift from cell counting toward integrated histologic assessment. AI-assisted quantification can achieve performance comparable to expert pathologists while enabling analysis of entire WSIs and assessment of spatial disease heterogeneity. However, reported performance should be interpreted in the context of overlapping datasets, as the CEGIR/OMEGA cohort was used by multiple models. Current barriers to widespread implementation include infrastructure requirements, differences in image acquisition and pathology workflows, and limited prospective validation in broader populations.

#### 4.4.2. Endoscopic Pattern Recognition

AI models trained on endoscopic white-light images showed promising results in detecting features of EoE. The AI-EoE algorithm was trained to distinguish EoE from a normal esophagus using endoscopic images. A second model, AI-EoE-EREFS, incorporated additional branches to identify individual EREFS features, allowing detection of specific endoscopic findings as well as binary disease classification. The AI-EoE-EREFS model was then tested on 200 white-light images from an external dataset at a different institution and achieved 96% sensitivity, 94% specificity, 95% accuracy, and an AUC of 0.992 for distinguishing EoE from normal esophagus. The model’s sensitivity was also significantly higher than that of senior fellows (96% vs. 75%) [52].

A separate CNN model was developed using ResNet50 architecture and trained on 1192 endoscopic images from 108 patients with histologically confirmed EoE and 1192 images of normal esophagus. For validation, the model was evaluated using 249 images from 39 endoscopic sessions in 35 patients with EoE and 507 images from 96 subjects with a normal esophagus. The model achieved 94.7% accuracy, 90.8% sensitivity, and 96.6% specificity on image-level analysis. At the case level, the model correctly identified 37 of 39 EoE cases, with 94.9% sensitivity and 99.0% specificity [53].

These systems also have the potential to support clinical decision-making during endoscopy. Real-time assistance may improve detection while also helping to standardize EREFS scoring and reduce interobserver variability. Despite these promising results, many AI models were trained on retrospective image datasets, and their performance in real-time clinical settings is still unknown. Differences in image acquisition and patient populations can also affect generalizability and introduce bias. Integration into existing endoscopy platforms represents a further practical barrier to widespread adoption. Further prospective and external validation are needed to assess model performance, calibration, and interpretability in real-world clinical settings. Overall, AI-based endoscopic analysis shows potential to improve EoE detection and standardize endoscopic assessment, but additional validation is needed before routine clinical use.

#### 4.4.3. Transcriptomic-Based Classification

AI approaches have expanded beyond endoscopic and histologic image analysis to include transcriptomics-based disease classification. In one study, a random forest model generated a diagnostic probability score for EoE (p(EoE)) based on esophageal mRNA transcript patterns from patients with EoE, GERD, and controls. In an external test set, a p(EoE) score of ≥25 distinguished EoE from non-EoE cases with 90.9% sensitivity, 93.2% specificity, and an AUC of 0.985. Among 13 patients with equivocal initial biopsy findings, the algorithm classified EoE versus GERD with 84.6% accuracy, with the eventual diagnosis established based on subsequent clinical follow-up [54]. These findings suggest that transcriptomic profiling may help classify patients with indeterminate histologic findings, but further external and prospective validation is needed before this approach can be incorporated into routine clinical practice.

Table 1 summarizes the clinical role, key study populations, reported diagnostic performance, validation status, and current applicability of the established and emerging diagnostic modalities discussed in this review.

## 5. Limitations Across Diagnostic Modalities

### 5.1. Lack of Standardization Across Modalities

A fundamental challenge in EoE diagnostics is the lack of standardization across testing modalities. From a histologic perspective, eosinophil counts are influenced by microscope high-power field size. Reporting eosinophil density as eos/mm^2^ has been proposed to address this issue, but its adoption in clinical practice has been limited [1]. Discordance also extends to endoscopic evaluation. EREFS scoring has been validated but is not consistently documented across centers, nor is it integrated into all reporting systems [55]. Emerging diagnostic tools such as Cytosponge, EST, mucosal impedance, and EndoFLIP face similar challenges. Differences in diagnostic thresholds and study protocols limit replicability and hinder broader clinical implementation [7].

### 5.2. Sampling Variability

Sampling variability remains a major limitation in EoE diagnosis. EoE is a patchy disease because eosinophilic infiltration is distributed unevenly throughout the esophagus. As a result, biopsy findings within the same patient can range from normal to markedly elevated across different biopsy sites [1]. This heterogeneity is further reflected in clinical data. In a study of 511 newly diagnosed patients, 56% had at least one biopsy site with fewer than 15 eos/hpf. Ten percent of these patients had non-distal disease that would have been missed with distal-only sampling [56]. The study concluded that routine biopsy protocols should include both distal and mid-esophageal sampling [56]. These findings differ from the current ASGE recommendations for biopsies from the mid or proximal esophagus [7]. These inconsistencies complicate both diagnosis and disease monitoring. False negatives in sampling can lead to underdiagnosis or premature discontinuation of therapy when assessing treatment response.

### 5.3. Cost and Accessibility

Endoscopy with biopsy is resource-intensive and requires sedation, specialized personnel, and pathologic processing [1]. As the incidence and prevalence of EoE continue to rise, the need for repeated procedures for disease monitoring places an increasing burden on both patients and healthcare systems [7]. Access to newer technologies is even more limited. Modalities such as EndoFLIP, mucosal impedance, and molecular diagnostics depend on specialized equipment that may be less widely available outside of tertiary centers. AI-based tools face similar challenges because their use requires computational resources that are not widely available. The cost-effectiveness of these emerging approaches has not yet been established in large-scale studies. Uncertainty about the value of these emerging technologies compared with standard endoscopy further complicates widespread adoption [7].

### 5.4. Validation and Generalizability Issues

Limitations in validity and generalizability remain a major barrier to the clinical adoption of emerging diagnostic tools. Most of the existing evidence is generated from single-center or small multicenter studies conducted at specialized referral centers with patient populations that may not reflect the clinical spectrum of EoE. This is particularly important in AI-based systems as these models rely on datasets for training. Variation in patient populations, training datasets, and healthcare resources may limit the generalizability of AI algorithms across different clinical environments [57]. Similar gaps also exist for biomarker-based approaches. Although blood-based biomarkers have been evaluated in adults, multi-marker approaches have been investigated more extensively in pediatric cohorts, which may limit the generalizability of these findings to adult populations [22,23,24]. Even highly accurate tools such as the EDP have only been validated in research settings, with external validation needed to further establish their clinical applicability [43].

Spectrum bias is an additional concern, as several studies evaluated diagnostic performance by comparing EoE with healthy or normal-esophagus controls rather than clinically relevant comparator populations such as GERD or non-EoE dysphagia. Such case–control designs may overestimate sensitivity and specificity compared with performance in patients presenting with similar symptoms and competing diagnoses.

### 5.5. Barriers to Clinical Adoption

Translating emerging diagnostic tools into routine clinical practice is a challenging process. Beyond issues of cost and accessibility, the deployment of AI-based diagnostics requires an appropriate regulatory framework to ensure clinical benefit while minimizing potential harm [57]. Integration of new technologies also requires meaningful changes in clinical workflows. This often necessitates additional training, adjustments in practice patterns, and investment in supporting infrastructure. Clinician familiarity and comfort with these new modalities may also be limited outside of specialty centers. Evidence gaps further complicate this process. The absence of prospective comparisons between emerging and conventional modalities makes it difficult to clearly define their role within diagnostic algorithms. The 2025 ACG guideline also acknowledges several of these tools but notes that modalities such as mucosal impedance and EndoFLIP remain largely restricted to specialized centers and no non-invasive biomarker is currently validated for clinical use [1].

## 6. Conclusions

The diagnostic evaluation of eosinophilic esophagitis has evolved substantially over recent decades. Traditional approaches centered on endoscopy and histologic assessment have expanded into a broader landscape that now includes HRM, EndoFLIP, mucosal impedance, non-endoscopic sampling techniques and molecular diagnostics.

Within this evolving diagnostic framework, artificial intelligence represents an important adjunct rather than a standalone solution. AI-driven approaches to histologic analysis and endoscopic interpretation have demonstrated high accuracy in retrospective and controlled datasets, with the potential to reduce interobserver variability and improve the efficiency of diagnostic assessment. As these technologies continue to develop, their role in standardized diagnostic pathways and longitudinal assessment will depend on further external validation.

Significant challenges restrict the implementation of many of these technologies. Variability in sampling, lack of standardization, and gaps in validation continue to limit widespread clinical application. Addressing these barriers will require multicenter validation studies, standardized diagnostic thresholds, and structured integration into clinical workflows. In a practical diagnostic and monitoring pathway, conventional EGD with biopsy remains the foundation for initial diagnosis and histologic assessment. For longitudinal monitoring, minimally invasive approaches such as TNE, Cytosponge, and the EST may reduce the need for repeated sedated procedures in selected patients, while blood-based biomarkers remain investigational. EndoFLIP and mucosal impedance may provide complementary structural and mucosal information during endoscopic assessment, while manometry may be considered when persistent dysphagia raises concern for a coexisting motility disorder. Tissue-based approaches such as EoEHSS, molecular assays, and digital pathology can provide additional information beyond conventional eosinophil quantification. AI-based tools may support automated histologic and endoscopic interpretation as prospective validation advances.

Looking forward, the greatest advances in EoE diagnosis will depend on the convergence of complementary approaches. The integration of structural, functional, molecular, and computational data offers the potential to improve diagnostic precision, personalize disease management, and enhance patient outcomes.

## Figures and Tables

**Table 1:** Comparative characteristics of emerging diagnostic and disease assessment modalities for EoE. The table summarizes the clinical role, study populations, reported diagnostic performance, validation status, and current clinical applicability of emerging approaches to EoE diagnosis and disease assessment.

Role	Modality	Population and Key Studies	Performance	Validation and Current Status
**Minimally** **Invasive** **Alternatives to EGD**	**TNE**	Pediatric and adult studies. 21 children [14]; 190 children/young adults (294 procedures) [15]; 24 adults (74 procedures) [17]	Vs EGD/biopsy; 98% procedure completion and 88–94% full-thickness epithelium in pediatric cohort [15]; 97% nasal intubation and adequate histology in completed adult procedures [17]	Prospective and retrospective pediatric/adult feasibility and comparative data [14,15,16,17]. Potential for serial assessment, but limited adult evidence and lamina propria sampling [15].
	**Cytosponge**	80 patients; 101 paired procedures in prospective two-center study [18]	Vs histologic biopsy: r = 0.78; sensitivity 75%, specificity 86% for ≥15 eos/hpf [18]	Prospective paired-biopsy validation. Minimally invasive, but sensitivity and processing requirements limit routine adoption.
	**EST**	134-patient cohort [19]	Vs histologic eosinophil density: AUC 0.83 for active vs. inactive EoE; combined biomarker score sensitivity 80%, specificity 75% for active EoE vs. inactive EoE or normal esophagus [19]	Clinical cohort data; no tissue histology. Primarily investigational.
	**TCE**	17 participants: 13 patients with EoE and 4 healthy controls [21]	Vs biopsy histology: visualization of intraepithelial eosinophils and agreement with histology; no formal diagnostic measures reported [21]	First-in-human feasibility data; broader mucosal coverage may reduce sampling limitations, but diagnostic accuracy and clinical utility for EoE remain unestablished.
	**Blood and** **Airway** **Biomarkers**	Multicenter cohort of 209 patients for AEC [22]. Pediatric multimarker studies [24]	EoE vs. non-EoE dysphagia: AUC 0.85; 155 eosinophils/mm^3^: sensitivity 87% and specificity 71%. Active EoE vs. histologic remission: AUC 0.705; 325 eosinophils/mm^3^: sensitivity 52.7% and specificity 82.3% [22]. Multimarker models: AUC up to 0.92 distinguishing active EoE, remission, and healthy states [24]	Heterogeneous cohorts and biomarker panels. No single biomarker validated for stand-alone diagnosis.
**Complementary Diagnostic** **Assessment**	**HRM**	Scoping review of 802 patients [27]. Comparative study of 21 EoE patients and 21 GERD patients [29].	EGJ outflow disorders ~5% across reviewed populations [27]. Panesophageal pressurization in 48% of EoE patients in one comparative cohort [29]	Not an EoE diagnostic test. Used selectively for persistent dysphagia or suspected coexisting motility disorder.
	**EndoFLIP**	C2D2 developed in 215 patients; 46-patient off-treatment subgroup [35]. Dysphagia cohort of 109 patients, including 29 with EoE [37]	Distensibility plateau < 225 mm^2^ (~17 mm) associated with future food impaction [33]; C2D2 ≤ 3 associated with histologic response to PPI (OR 14.5; 95% CI, 2.6–85) [35]; 25/29 EoE patients had reduced distensibility [37]	Complementary structural/functional assessment. Guidance supports use when persistent dysphagia remains unexplained after endoscopy/histopathology. Wide CI and retrospective treatment-escalation data limit interpretation [35,37].
	**Mucosal** **Impedance**	91 retrospective discovery and 49 prospective validation patients [38] 69-patient prospective balloon MI study [39] 30-patient active/inactive/control study [40]	EoE vs. non-EoE with dysphagia (histologic diagnosis): sensitivity 100%, specificity 96% in prospective validation [38] EoE identification among GERD and non-GERD: Balloon MI AUC 0.89 [39] Active EoE vs. inactive EoE and controls: MI < 2300 Ω sensitivity 90%, specificity 91% [40]	Includes prospective and multicenter validation, but cohorts remain relatively small and thresholds/protocols vary. Limited clinical availability.
**Tissue-Based** **Assessment**	**EoEHSS**	201 biopsy samples from 104 subjects in initial validation study [42]	Treatment status: EoEHSS principal components AUC 77.8% vs. 69.8% for distal peak eosinophil count; Moderate-to-strong interobserver agreement [42]	Initial validation data; greater complexity and limited adoption support complementary rather than replacement use.
**Molecular** **Assessment**	**EDP**	Development: 29 pediatric samples Validation: 194 pediatric/adult samples [43]	EoE vs. Healthy Controls (histologic diagnosis): ~96% sensitivity, ~98% specificity [43]	Separate development and validation cohorts, including fresh and FFPE tissue. Specialized molecular testing; investigational.
	**Molecular** **Endotyping and Transcriptomics**	Studies defining EoEe1/e2/e3 and additional type 2 molecular subgroups [45,46]	Molecular endotypes independent of peak eosinophil count and stable across testing methods [45]	Primarily research-based; potential for molecular phenotyping and precision medicine. Broader clinical validation needed [45,46,47].
**AI and Digital** **Pathology**	**Automated** **Eosinophil Quantification**	203 patients with mucosal eosinophilia [48]; 289 WSIs from 183 pediatric patients [49]; 1066 images from 400 CEGIR/OMEGA patients [50]	Vs pathologist assessment: AUC 0.98 for >15 eos/hpf [48] Vs pathologist assessment: 91% accuracy for ≥15 eos/hpf [49] EoE Disease Activity: Sensitivity 94.13%, specificity 95.25% for disease activity [50]	Predominantly retrospective datasets; some overlapping CEGIR/OMEGA cohorts. External/prospective validation and workflow integration needed.
	**AI Endoscopic** **Pattern** **Recognition**	AI-EoE-EREFS: 200 WLI images [52]; ResNet50: 2384 training images and 756 validation images from 35 EoE patients and 96 controls [53].	Vs histologic diagnosis: AI-EoE-EREFS sensitivity 96%, specificity 94%, AUC 0.992 [52]. Vs histology confirmed EoE/normal esophagus: Independent ResNet50 image sensitivity 90.8%, specificity 96.6% [53]	High performance in retrospective image datasets. External/prospective validation, calibration, population diversity, and workflow integration remain necessary.
	**Transcriptomic** **Classification**	Training cohort of 113 patients Equivocal set of 13 patients [54]	EoE vs. non-EoE: sensitivity 90.9%, specificity 93.2%, AUC 0.985; Equivocal cases: 84.6% accuracy for EoE vs. GERD [54]	Research-stage approach. Broader prospective and external validation needed.

## Data Availability

No new data were created or analyzed in this study. Data sharing is not applicable to this article.
